# Fatigue 5 years after treatment for rectal cancer – results from the QoLiRECT study

**DOI:** 10.2340/1651-226X.2026.46460

**Published:** 2026-09-18

**Authors:** Johan Ekstrand, Rode Grönkvist, Carolina Ehrencrona, Elisabeth Gonzalez, Jacob Rosenberg, Eva Haglind, Eva Angenete

**Affiliations:** a Department of Surgery, SSORG – Scandinavian Surgical Outcomes Research Group, Institute of Clinical Sciences, Sahlgrenska Academy, University of Gothenburg, Gothenburg, Sweden; b Department of Surgery, Växjö Hospital, Region Kronoberg, Växjö, Sweden; c School of Public Health and Community Medicine, Institute of Medicine, Sahlgrenska Academy, University of Gothenburg, Gothenburg, Sweden; d Department of Surgery, Herlev Hospital, University of Copenhagen, Copenhagen, Denmark

**Keywords:** Rectal neoplasms/surgery, rectal neoplasms, therapy, fatigue, quality of life, risk factors, surveys and questionnaires, Sweden/epidemiology, Denmark/epidemiology

## Abstract

**Background and purpose:**

Fatigue affects colorectal cancer survivors and has a significant impact on overall quality of life (QoL) and possibly overall survival. Fatigue, with focus on patients with rectal cancer, is less studied. The aim of this study was to investigate fatigue prevalence at 5 years after rectal cancer surgery.

**Patient/material and methods:**

Data were collected from the Quality of Life in RECTal cancer study, a prospective longitudinal multicenter study of patients with rectal cancer, also using data from Swedish and Danish colorectal cancer registries. Baseline characteristics and treatment-related factors were investigated as potential predictors for fatigue measured with the Piper Fatigue Scale (PFS). Patients were included at diagnosis and answered questionnaires at baseline and at 5 years after diagnosis.

**Results:**

The cohort consisted of 1024 patients who underwent surgery for rectal cancer. Five hundred and ninety three patients responded to the 5-year follow-up questionnaire. At 5 years, 52.4% of patients experienced fatigue. Moderate-severe fatigue (PFS ≥ 4) was reported by 172 patients (29%). Higher Body mass index (BMI) at baseline was associated with PFS ≥ 4 (Odds ratio [OR] 1.07, 95% Confidence interval [CI] 1.02–1.11, *p* = 0.004) in the univariate analysis, with similar results in multivariate analysis. A significant association was found between pain and moderate-to-severe fatigue in univariate analysis, OR 4.34 (*p* < 0.0001, 95% CI 2.91–6.49), but not in multivariate analysis, 2.8 (*p* < 0.0001, 95% CI 1.79–4.58). Higher QoL was negatively correlated with PFS ≥ 4 in both uni- and multivariate analyses (OR 0.37, 95% CI 0.30–0.46, *p* < 0.0001; OR 0.34, 95% CI 0.27–0.44 *p* < 0.0001). Major low-anterior resection syndrome (OR 2.17, 95% CI 1.09–4.34, *p* = 0.028) and permanent stoma (OR 1.92, 95% CI 1.06–3.47, *p* = 0.032) were associated with PFS ≥ 4 in the univariate, but not in the multivariate, analysis.

**Interpretation:**

Moderate-severe fatigue is prevalent in rectal cancer survivors. Targeted prevention of fatigue might be of interest in long-term rehabilitation of rectal cancer survivors.

## Introduction

In patients diagnosed with rectal cancer, both patient-specific and treatment-related factors may influence long-term quality of life (QoL). As survival improves and early-onset colorectal cancer becomes more frequent [1], identification of patients at risk for developing symptoms that can affect long-term QoL is necessary to improve functional outcomes. Fatigue is considered one of the most complex and prevalent symptoms affecting overall health-related QoL [2]. Although a universally accepted definition is lacking, the National Comprehensive Cancer Network guidelines define fatigue as ‘a distressing, persistent, subjective sense of physical, emotional, and/or cognitive tiredness or exhaustion related to cancer and/or cancer treatment that is not proportional to recent activity and interferes with usual functioning’ [3]. In the general population, about 10% experience chronic fatigue lasting defined as fatigue lasting more than 6 months [4]. Fatigue is more prevalent in women than in men, both globally and in the Swedish community [5]. In colorectal cancer survivors, almost 40% have been reported to experience long-term fatigue, also suggesting that fatigue has a significant impact on QoL in these patients. In addition to affecting patient mood, social activities, and work ability [6], fatigue may negatively influence survival [7].

Patients with different types of cancer are at different risks for fatigue development [8, 9]. As colon and rectal cancer differ in functional outcome after treatment and little is known about long-term fatigue in patients with rectal cancer, it is important to study this population separately. In patients with rectal cancer, a few factors at the time of diagnosis have been identified as possible predictors for post-treatment fatigue, such as female sex [10] and obesity [11]. Treatment-related factors include neoadjuvant treatment [12, 13] and surgical technique, with robot-assisted surgery being associated with less fatigue than traditional laparoscopy [14]. After treatment, low-anterior resection syndrome (LARS) [15] increases the risk of short-term fatigue development. However, factors affecting long-term fatigue, as well as its prevalence in patients with rectal cancer, have been studied less extensively. If early identification of patients at increased risk for developing fatigue could be performed, initiation of preventive measures, such as recommendations for physical activity, could be emphasized [16, 17].

The aim of this study was to explore the prevalence of fatigue 5 years after surgery for rectal cancer and to examine how baseline patient characteristics and treatment-related factors correlated with patient-reported fatigue.

## Patients/material and methods

### Data collection

The Quality of Life in RECTal cancer (QoLiRECT) study was a prospective observational multi-center study of QoL and functional outcomes in patients with rectal cancer (ClinicalTrials.gov, NCT01477229). A total of 1248 patients from Sweden and Denmark were enrolled in the study between 2012 and 2015. Study inclusion and exclusion criteria have been described elsewhere [18]. Fatigue-related questions were only included in the 5-year questionnaire. Analyses of predictors were based on data collected from the QoLiRECT questionnaires as well as clinical data acquired from the Swedish and Danish national quality registers for colorectal cancer. The original study population included 1215 patients. At baseline, the present substudy sample included 1024 subjects meeting the inclusion criteria: low anterior resection, Hartmann’s procedure, or abdominoperineal excision, and 1085 patients answered the baseline questionnaire, and 593 patients responded to the 5-year questionnaire.

### The Piper Fatigue Scale

The 22-item Revised Piper Fatigue Scale (rPFS) is used to assess cancer-related fatigue [19] and has been extensively validated. The rPFS consists of 22 numerically scaled items measuring four different dimensions/subscales of subjective fatigue: behavioral/severity, affective meaning, sensory, and cognitive/mood. These items were incorporated in the 5-year questionnaire within the QoLiRECT study, using a previous Swedish translation [20] and a study-specific Danish version. The translated rPFS in the questionnaire has some notable differences from the original rPFS. In the questionnaire, the response category ‘0’ (no fatigue) was removed, so the responses ranged from 1 to 10. In addition, an opening item was included requesting respondents to rate their overall fatigue on a scale from 1 to 10. If the answer was ‘1’, indicating no fatigue, respondents were instructed to skip all subsequent questions concerning fatigue. Missing values were handled by mean item substitution, as described [19]. From the mean values of the PFS subscales, a total fatigue score is calculated, ranging from 1 to 10 and graded as none (1), mild (2–3), moderate (4–6), or severe (7–10).

### Assessment of predictors for fatigue development

To investigate potential preoperative (baseline) predictors of moderate-to-severe fatigue at 5 years after treatment for rectal cancer, logistic regression was performed using both univariate and multivariate analyses. Based on clinical expertise and previous publications, age, sex, BMI, smoking, and alcohol intake at baseline were used as patient-specific preoperative predictors for fatigue development. For treatment-specific and postoperative predictors, tumor stage (Union for International Cancer Control tumor stage; UICC), tumor height (affecting the extent of mesorectal excision and surgical technique), neoadjuvant treatment, surgical treatment (low anterior resection, abdominoperineal excision, and Hartmann’s operation), permanent stoma, multivisceral resection, and LARS prevalence, patient-perceived pain and overall QoL at 5 years postoperatively were used. Data for risk factor assessment were collected both from the QoLiRECT questionnaires at baseline (smoking, alcohol intake) and at 5 years. In the QoLiRECT 5-year questionnaire, pain was assessed using a 11-point Likert scale where 0 was no pain and 10 was the worst pain felt during the last 24 hours. Overall QoL was assessed using a 7-grade Likert scale. LARS score was estimated in the 5-year questionnaire as previously described [21]. Data on age, sex, treatment-related factors, and postoperative predictors were collected from the Swedish and Danish national colorectal cancer registries. For missing values in the investigated predictors, multiple imputations by chained equations (MICE) were performed. A total of 50 imputations with 10 iterations were used. In the predictor matrix, postoperative covariates were not used to impute preoperative variables. The primary outcome, fatigue at 5 years, was used to impute all covariates.

## Results

### Demographic analysis

Demographic data and variable levels for the 1024 patients are given in Table 1. Demographic and clinical characteristics were generally comparable between baseline and 5-year respondents. However, UICC tumor stages 3–4 were somewhat more common in the baseline cohort. Conversely, patients with UICC stage 1 rectal cancer constituted a higher proportion of the 5-year respondent group. Data on LARS prevalence in patients with bowel continuity, pain, and QoL, as well as permanent stoma prevalence, are shown only for 5-year respondents.

**Table 1:** Demographic characteristics at baseline and at 5 years after surgery for rectal cancer.

Baseline Characteristics	Overall (*N* = 1024)	5-year follow-up (*N* = 593)
**Age**		
Mean (SD)	67.3 (10.4)	65.9 (9.74)
Median [Min, Max]	69.0 [19.0, 91.0]	67.0 [25.0, 91.0]
**Sex**		
Male	645 (63.0%)	368 (62.1%)
Female	379 (37.0%)	225 (37.9%)
**BMI**		
Mean (SD)	26.0 (4.35)	25.8 (4.02)
Median [Min, Max]	25.5 [15.5, 55.5]	25.4 [16.7, 42.4]
Missing	4 (0.4%)	0 (0%)
**Smoker (currently or previously (at baseline))**		
No	335 (32.7%)	214 (36.1%)
Yes	582 (56.8%)	358 (60.4%)
Missing	107 (10.4%)	21 (3.5%)
**Alcohol consumption (drinks per week, at baseline)**		
I don’t drink alcohol	192 (18.8%)	102 (17.2%)
1–2	294 (28.7%)	188 (31.7%)
3–4	189 (18.5%)	119 (20.1%)
5–10	143 (14.0%)	105 (17.7%)
11–15	49 (4.8%)	28 (4.7%)
> 16	34 (3.3%)	22 (3.7%)
Missing	123 (12.0%)	29 (4.9%)
**UICC Stage**		
1	337 (32.9%)	228 (38.4%)
2	246 (24.0%)	151 (25.5%)
3	339 (33.1%)	176 (29.7%)
4	87 (8.5%)	31 (5.2%)
Missing	15 (1.5%)	7 (1.2%)
**Tumor height**		
Low (0–5 cm)	197 (19.2%)	117 (19.7%)
Middle (5–10 cm)	372 (36.3%)	213 (35.9%)
High (> 10 cm)	435 (42.5%)	249 (42.0%)
Missing	20 (2.0%)	14 (2.4%)
**Neoadjuvant radiotherapy**		
No	419 (40.9%)	235 (39.6%)
Yes	594 (58.0%)	354 (59.7%)
Missing	11 (1.1%)	4 (0.7%)
**Neoadjuvant chemotherapy**		
No	803 (78.4%)	471 (79.4%)
Yes	210 (20.5%)	118 (19.9%)
Missing	11 (1.1%)	4 (0.7%)
**Surgical treatment**		
Anterior resection	551 (53.8%)	349 (58.9%)
Hartmann´s operation	100 (9.8%)	38 (6.4%)
Abdominoperineal excision	373 (36.4%)	206 (34.7%)
**Low anterior resection syndrome (LARS, at 5-year follow-up)**		
Patients with stoma		289 (48.7%)
Patients without stoma		280 (47.2%)
No LARS		68 (24.3%)
Minor LARS		65 (23.2%)
Major LARS		147 (52.5%)
Missing		24 (4.0%)
**Reporting pain (5-year follow-up)**		
No		386 (65.1%)
Yes		162 (27.3%)
Missing		45 (7.6%)
**Quality of life (5-year follow-up, 0–6)**		
Mean (SD)		4.60 (1.05)
Median [Min, Max]		5.00 [1.00, 6.00]
Missing		3 (0.5%)

UICC: Union for International Cancer Control.

### Fatigue at 5 years after rectal cancer

Subscale and total PFS scores among patients in the 5-year cohort that reported experiencing fatigue to any extent are described in Table 2. Among 5-year respondents, 311 of 593 (52.4%) reported experiencing fatigue to some extent. Among patients who experienced fatigue, the mean (SD) fatigue scores for the different subscales were 4.3 (2.2) (behavioral severity), 4.9 (2.0) (affective meaning), 4.9 (1.9) (sensory), and 4.0 (1.8) (cognitive/mood). The total PFS score was 4.5 (1.8). Moderate-to-severe fatigue, as indicated by a total PFS score of ≥ 4, was reported by 172 patients (29.0%).

**Table 2:** Prevalence of fatigue at any extent 5 years after treatment for rectal cancer.

PFS subscale	Mean (SD)	Median (95% CI)	Missing *n*, (%)
Total PFS score	4.5 (1.8)	4.7 (4.4–4.9)	34 (10.9)
Behavioral/severity	4.3 (2.2)	4.2 (3.7–4.4)	23 (7.4)
Affective/meaning	4.9 (2.0)	5.0 (4.6–5.0)	24 (7.7)
Sensory	4.9 (1.9)	5.2 (4.8–5.4)	19 (6.1)
Cognitive/mood	4.0 (1.8)	4.0 (3.5–4.2)	19 (6.1)

PFS: Piper Fatigue Scale.

### Preoperative associative predictors of fatigue development

**Figure 1:**
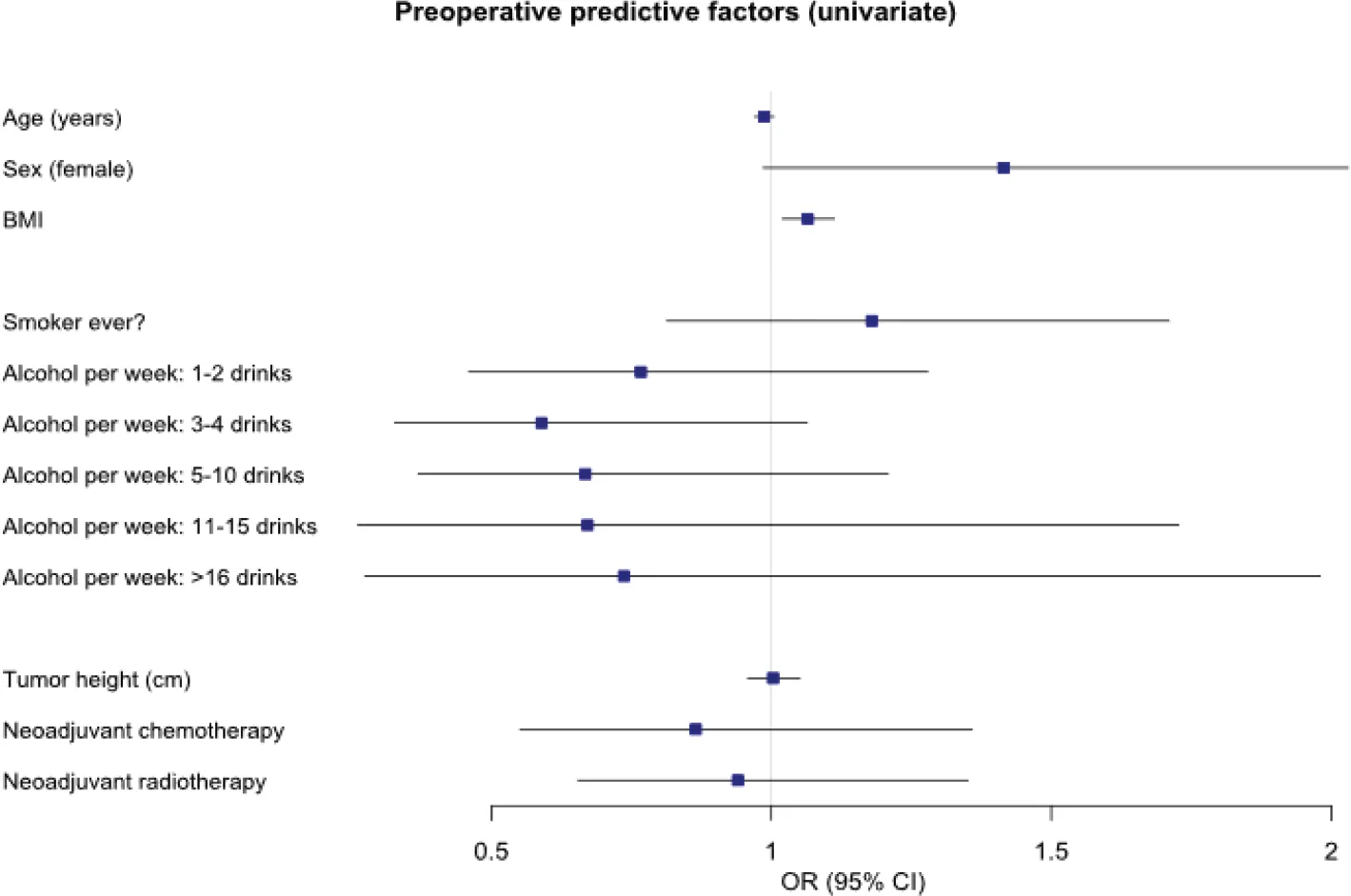
Univariate analysis of preoperative baseline candidate predictors of moderate-to-severe fatigue (PFS ≥ 4) at 5 years after treatment for rectal cancer. PFS: Piper Fatigue Scale.

**Figure 2:**
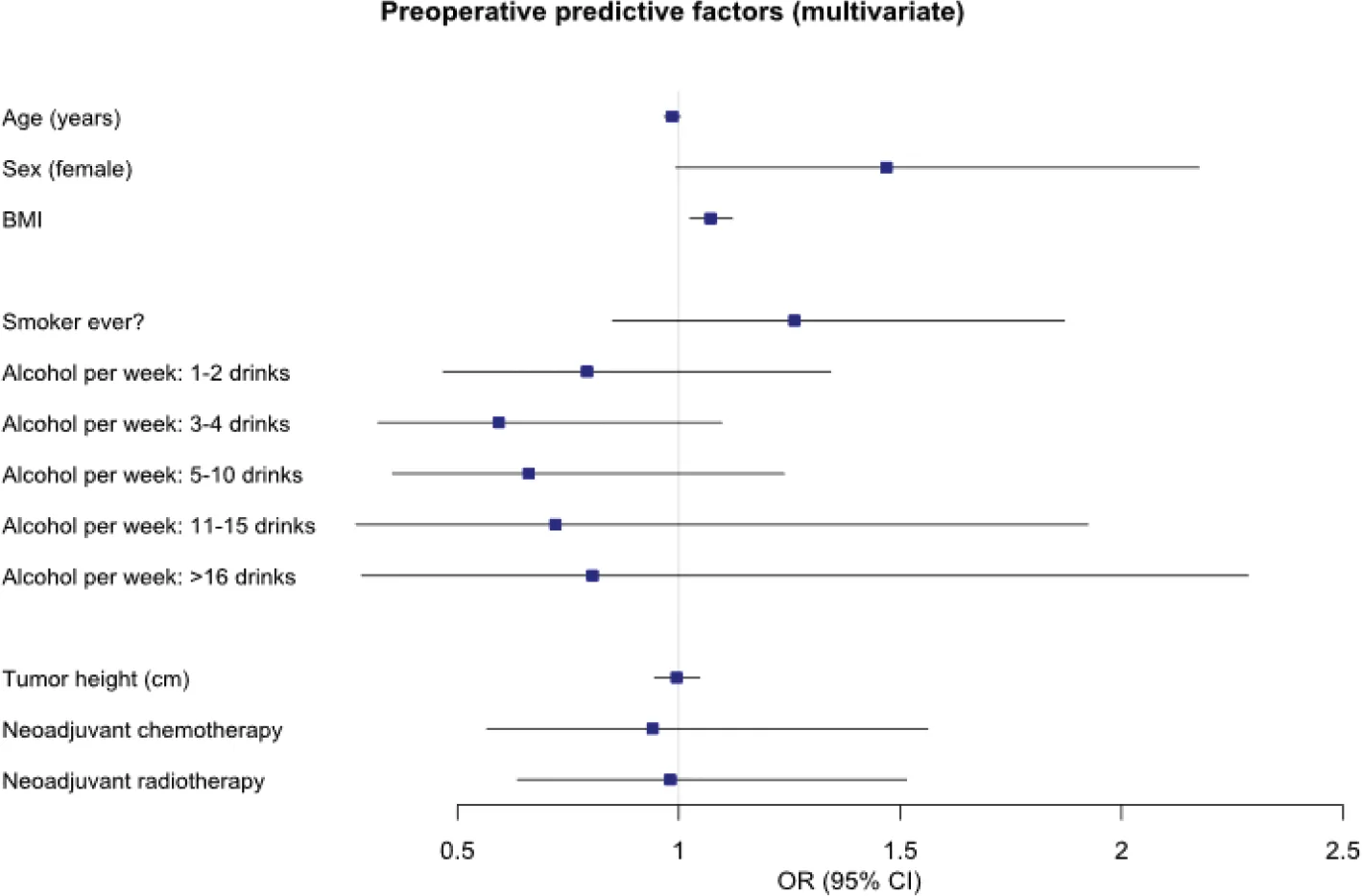
Multivariate analysis of preoperative baseline candidate predictors of moderate-to-severe fatigue (PFS ≥ 4) at 5 years after treatment for rectal cancer. PFS: Piper Fatigue Scale.

The outcome across all models was the total PFS score, with levels of ‘no or mild fatigue’ (PFS total score < 4) versus ‘moderate-to-severe fatigue’ (PFS total score ≥ 4). BMI at baseline was significantly associated with moderate-to-severe fatigue at 5 years, with a similar OR of 1.07 and 95% CI (1.02–1.11) in both the univariate (*p* = 0.004) and multivariate (*p* = 0.002) analyses. There was a trend towards an association between female sex and PFS ≥ 4 in both univariate and multivariate analyses, with an OR of 1.47 and 95% CI (0.99–2.17) and OR 1.41 and 95% CI (0.99–2.03), respectively. However, statistical significance was not reached for female sex as a predictor of fatigue. No association with other factors such as age, smoking, alcohol intake, and neoadjuvant treatment was seen in either the univariate or the multivariate analyses (Figures 1 and 2).

### Postoperative associative predictors of fatigue development

**Figure 3:**
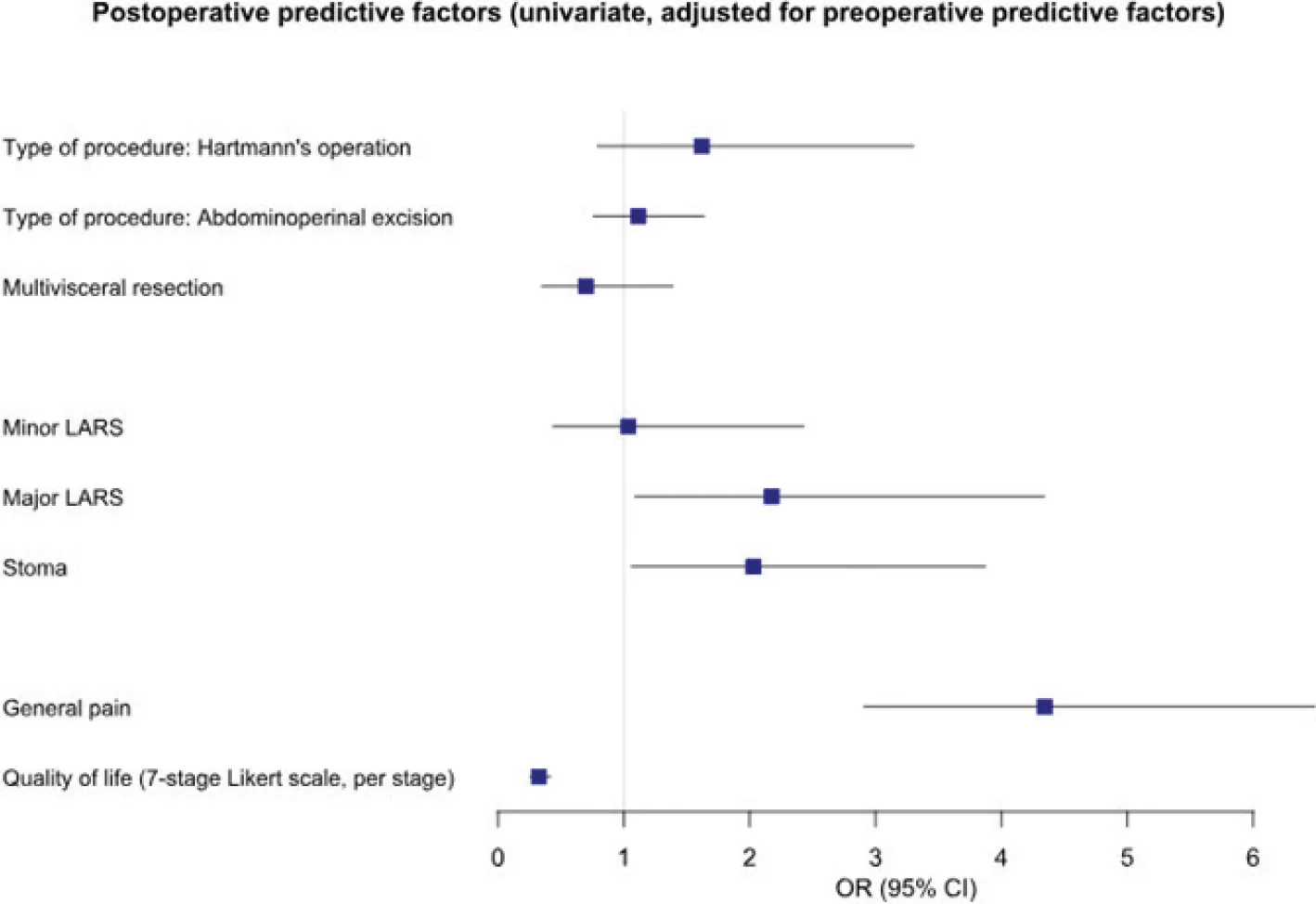
Univariate analysis of postoperative candidate predictors of moderate-to-severe fatigue (PFS ≥ 4) at 5 years after treatment for rectal cancer. PFS: Piper Fatigue Scale; LARS: low anterior resection syndrome.

**Figure 4:**
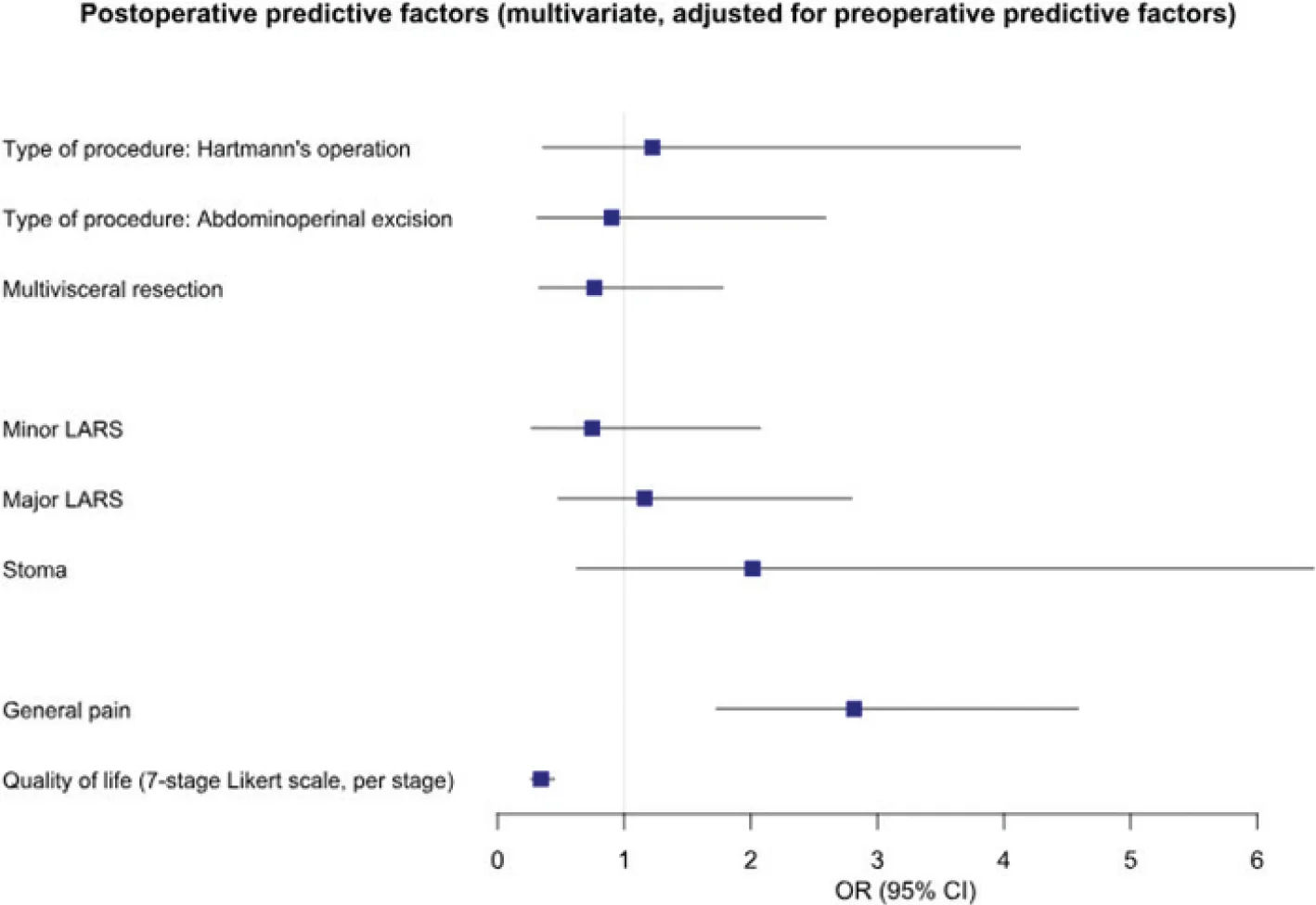
Multivariate analysis of postoperative candidate predictors of moderate-to-severe fatigue (PFS ≥ 4) at 5 years after treatment for rectal cancer. PFS: Piper Fatigue Scale; LARS: low anterior resection syndrome.

Postoperative associative predictors of PFS ≥ 4 were investigated in univariate and multivariate analyses. These analyses were also adjusted for preoperative predictors; both univariate and multivariate analyses found a significant association between pain and moderate-to-severe fatigue. In univariate analysis, the OR was 4.34 (*p* < 0.0001, 95% CI 2.91–6.49), and in multivariate analysis, 2.8 (*p* < 0.0001, 95% CI 1.79–4.58). A negative correlation for moderate-to-severe fatigue was found with increasing QoL. Each increasing point on a seven-grade scale measuring QoL at 5 years was associated with a lower risk for PFS ≥ 4 with OR 0.37 (*p* < 0.0001, 95% CI 0.30–0.46) in the univariate analysis and OR 0.34 (*p* < 0.0001, 95% CI 0.27–0.44) in the multivariate analysis. Major LARS and permanent stoma were both associated with moderate-to-severe fatigue in the univariate analysis with ORs of 2.17 (*p* = 0.028, 95% CI 1.09–4.34) and 1.92 (*p* = 0.032, 95% CI 1.06–3.47), respectively. However, multivariate analysis failed to demonstrate a significant association between major LARS and permanent stoma, with a respective OR of 1.16 (*p* = 0.74, 95% CI 0.48–2.79) and 2.01 (*p* = 0.24, 95% CI 0.63–6.45) (Figures 3 and 4).

## Discussion and conclusion

The present study found a high prevalence of fatigue in patients alive 5 years after treatment for rectal cancer. An association between moderate-to-severe fatigue, defined as a PFS total score ≥ 4, and increasing BMI at the time of diagnosis is demonstrated. Pain at 5 years postoperatively was associated with a PFS score ≥ 4, and there was also a correlation between increasing fatigue and lower QoL. Furthermore, we found that major LARS and the presence of a permanent stoma were associated with moderate-to-severe fatigue at 5 years postoperatively, albeit only significant in the univariate analysis. The prevalence of chronic fatigue in the general population is estimated to be around 10% globally [4], and our results suggest that more than half of patients with rectal cancer suffer from some degree of fatigue at 5 years after treatment, and almost one third of patients experience moderate-to-severe fatigue. This is important as many studies have indicated that fatigue has a strong negative impact on overall QoL [22, 23], a finding that was confirmed in this study as a strong correlation between increasing QoL and less moderate-to-severe fatigue was demonstrated.

Among the PFS subscales, the highest scores in the study cohort were observed for affective and sensory fatigue. This could be translated into what other studies refer to as mental rather than physical fatigue, although the two are often strongly interconnected [24]. We found that LARS, as well as the presence of a stoma, seemed related to fatigue, although not significant in the multivariate analysis. Possibly, physical dysfunction causes psychological discomfort that, in turn, affects QoL. We have previously found that bother of physical functions such as bowel function has a negative effect on QoL [25]. Our data corroborate previous data on the association between LARS and increased fatigue [26]. Since there is no standard treatment for LARS [27], a multimodal approach to these patients is mandated. Our results suggest that increased attention to LARS and measures such as preoperative counselling [28] may improve overall QoL in patients with rectal cancer by also reducing fatigue. To prevent fatigue, pre-habilitation before surgery and oncological treatment and lifestyle modifications such as increasing physical activity may be used [29]. While the favorable effect of physical exercise on fatigue in colorectal cancer survivors has previously been questioned [30, 31], recent data suggest that physical exercise in the perioperative period in patients with colorectal cancer reduces the development of fatigue up to 12 months postoperatively [16].

Other lifestyle factors that could be addressed are obesity and alcohol consumption. In our study, increasing BMI was significantly associated with moderate-to-severe fatigue. While there is an established link between obesity and fatigue [32], the mean BMI in the study cohort did not indicate a high prevalence of obesity. Our results therefore suggest that increasing BMI can affect fatigue, regardless of obesity or not. In-depth studies of long-term fatigue and QoL in obese patients are of interest, particularly since obesity may be associated with longer survival in stage IV colorectal cancer [33]. No significant association with moderate-to-severe fatigue was demonstrated regarding different levels of alcohol intake. In the postoperative multivariate model, alcohol consumption (3–4 units/week) was statistically significant only as an adjustment factor (data not shown). However, because this variable became significant only as an adjustment factor in a model that included postoperative data, it could not be interpreted as a significant baseline predictor of fatigue. A recent study did, however, demonstrate that moderate alcohol consumption in patients with colorectal cancer was associated with less anxiety, less depression, and higher overall QoL up to 2 years after treatment [34]. While numerous studies have shown that female sex is associated with increased fatigue in patients with colorectal cancer [10, 35], female sex did not reach statistical significance as a predictor of moderate-to-severe fatigue in our study. However, a strong trend towards significance was seen, suggesting an association between female sex and moderate-to-severe fatigue. The female subgroup represented roughly one third of the study population. This limited sample size may reduce statistical power and thereby make it more difficult to detect a significant association even if it exists. Interestingly, neoadjuvant treatment did not affect fatigue at 5 years in our study. Previous studies do, however, suggest an impact at earlier timepoints [12, 13], indicating that fatigue induced by neoadjuvant treatment may, to some extent, improve over time.

The strengths of this study include the longitudinal design as well as long follow-up. The use of a validated fatigue questionnaire is also important. Another strength includes the multicenter design that increases generalizability. Study limitations include the lack of baseline data from the fatigue questionnaire. Our predictors chosen for the multivariate analysis did not include all factors that could be of importance, such as education and sleeping habits. Some of these factors could be extracted from the questionnaire but were omitted due to model complexity and sample size constraints. There was also substantial patient loss during the long follow-up period. However, in a cohort of patients with rectal cancer, with a majority of patients over 70 years of age, this is not entirely surprising. An additional limitation is that we cannot exclude that the prevalence of fatigue may be underestimated and prediction effects biased due to selective response associated with varying health at long-term follow-up. It is also a limitation that we do not have a cohort to compare with, apart from historical data.

In conclusion, we found that around half of patients experienced fatigue 5 years after treatment for rectal cancer, and almost a third of patients reported moderate-to-severe fatigue symptoms. This is one of the first studies that present the prevalence of fatigue 5 years after treatment for rectal cancer. Further attention to this complex symptom and its impact on QoL is warranted. It is possible that focusing on lifestyle factors as well as treatment for functional problems after treatment could reduce fatigue and thus improve long-term QoL in patients diagnosed with rectal cancer.

## Data Availability

Request to access the original data should be made to the corresponding author and will be considered based on the context of the request
